# Small internal jugular veins with restricted outflow are associated with severe multiple sclerosis: a sonographer-blinded, case–control ultrasound study

**DOI:** 10.1186/1471-2377-13-90

**Published:** 2013-07-17

**Authors:** Željko Krsmanović, Maja Živković, Toplica Lepić, Aleksandra Stanković, Ranko Raičević, Evica Dinčić

**Affiliations:** 1 Department of Neurology, Military Medical Academy, Belgrade 11 000, Serbia; 2 Laboratory for Radiobiology and Molecular Genetics, Institute of Nuclear Sciences “Vinča”, Belgrade 11 000, Serbia

**Keywords:** Multiple sclerosis, Echo-color Doppler, EDSS, CCSVI, Small internal jugular veins, Severity

## Abstract

**Background:**

Recent evidence has indicated an association between chronic cerebrospinal venous insufficiency (CCSVI) and multiple sclerosis. Small internal jugular veins (IJVs) (with a cross-sectional area of less than 0.4 cm^2^) have been previously described as difficult to catheterize, and their presence may potentially affect cerebrospinal venous drainage. In this blinded extracranial color-Doppler study we had two principal aims: first, to assess prevalence of CCSVI among Serbian MS patients compared to healthy controls; and second, to assess prevalence of small IJVs (with a CSA ≤ 0.4 cm^2^) among MS patients and controls.

**Methods:**

The sixty seven unrelated patients with clinical isolated syndrome (CIS), relapsing-remitting (RR), secondary progressive (SP) and primary progressive (PP) multiple sclerosis and 21 healthy controls were examined by high-resolution color-Doppler.

**Results:**

The ultrasonographic criteria of CCSVI (according to Zamboni) were positive in 11.9% of the patients and in none of the control subjects. The CCSVI-positive patients had significantly longer disease durations and were significantly more disabled (measured by their Expanded Disability Status Scale (EDSS) and Multiple Sclerosis Severity Score (MSSS) scores), but after adjustment for gender and disease duration, CCSVI was not an independent risk factor for multiple sclerosis severity. The small IJVs were found in 28.4% of the patients and 28.6% of the controls. The patients with small IJVs were associated with decreased venous outflow from the brain and presented with longer disease durations and significantly higher EDSS and MSSS scores compared to patients without small IJVs. A multivariate logistic regression analysis adjusted for gender and disease duration showed that small IJV is an independent factor associated with multiple sclerosis severity (EDSS ≥6) (adjusted OR = 8.9, 95% CI: 1.8-45.6, p = 0.007). Among patients with small IJVs the 36.84% were also CCSVI positive.

**Conclusions:**

Both, CCSVI and small IJVs seem to influence or follow MS severity, but only small IJVs turned out to be an independent factor in this study. Thus, small IJVs with restricted outflow, which might be aspects of CCSVI different from the criteria originally described by Zamboni, emerge as a cofactor in the multifactorial pathophysiology of multiple sclerosis.

## Background

Multiple sclerosis (MS) is a chronic autoimmune disease of the central nervous system (CNS). This disease is characterized by inflammation, demyelination and axonal injury, which leads to the formation of sclerotic plaques [1]. The complex etiology and heterogeneity of clinical outcomes associated with MS have led investigators to search for new risk factors and etiological explanations for the development and progression of this disease. One of the proposed factors was chronic cerebrospinal venous insufficiency (CCSVI) [2], which is characterized by anomalies of the main extracranial cerebrospinal (CS) venous routes (internal jugular veins (IJVs), the vertebral veins (VVs) and the azygos vein (AZY). The conflicting results about association between CCSVI (and its clinical correlates) and MS have been presented [2-6] and the question emerged if it is a primary or secondary condition to MS. It is believed that noninvasive venous echo-color ultrasound and transcranial (ECD/TCD) Doppler is useful for revealing the presence of outflow disturbances and morphological abnormalities in IJVs and VVs [7,8]. Using differing methodologies, all of these studies showed that IJV stenosis, defined either as a cross-sectional area (CSA) ≤ 0.3 cm2 or as a local CSA reduction of ≥50% [2,4,9], is one of the most frequently observed CCSVI criteria in patients with MS. IJV asymmetries were first observed in healthy subjects. Approximately one-third of healthy adults (34%) have one IJV that is significantly smaller than the other [10]. In 2001, prior to the research linking CCSVI to MS, Lichtenstein et al. examined IJV asymmetry among intensive care unit patients undergoing catheterization of the IJV as a routine procedure [11]. These researchers observed that 23% of IJVs were small, with a CSA ≤ 0.4 cm^2^, which could complicate the catheterization procedure [11]. However, neither them nor others have examine whether an IJV CSA ≤ 0.4 could be associated with MS, either as a primary or secondary phenomenon. There were two principal aims of this study: first, to assess prevalence of CCSVI among Serbian MS patients compared to healthy controls; and second, to assess prevalence of small IJVs (with a CSA ≤ 0.4 cm^2^) among MS patients and controls.

## Methods

### Ethics statement

The Ethical Committee of the Military Medical Academy (MMA), Belgrade, Serbia, approved this study. Each participant gave written informed consent to participate in the study.

### Study design

This cross-sectional study aims to assess prevalence of CCSVI and prevalence of small IJVs (with a CSA ≤ 0.4 cm^2^) among MS patients and controls from Serbia and to evaluate its correlation with clinical parameters of disease severity. The sixty seven unrelated patients with clinical isolated syndrome (CIS), relapsing-remitting (RR), secondary progressive (SP) and primary progressive (PP) multiple sclerosis, of Serbian origin, were recruited from the Neurology Clinic of the MMA, Serbia. All patients except CIS fulfilled the criteria for clinically definite MS [12] and the course of the disease was determined based on clinical data [13]. The chronic progressive (CP) group included the SP and PP MS patients. Disease severity was estimated using the Multiple Sclerosis Severity Score (MSSS) [14], which represents the Expanded Disability Status Scale (EDSS) [15] corrected for disease duration. Global MSSS values were calculated according to clinical data at the same time when ultrasound measurements were performed. The patients included in the study were never receiving a disease modifying therapy. On the basis of EDSS value the patients were divided into those with lower functional disability (EDSS < 6) and those with high disability (EDSS ≥ 6). The patient group consisted of 32 females and 35 males, of mean age of 35.7 ± 9.5 years and mean disease age at onset 27.6 ± 7.5 years (Table 1). The control group consisted of 7 female and 14 male healthy volunteers of mean age of 32.9 ± 10.1 years. The controls were of the same ethnical origin as the MS patients.

**Table 1:** Characteristics of patients with MS and controls

**Parameter**	**Patients (n = 67)**	**Controls (n = 21)**	**p**
Female/male (%)	48.0/52.0	33.3/66.7	ns*
Age (years ± SD)	35.7 ± 9.5	32.9 ± 10.1	ns^‡^
Age at onset (years ± SD)	27.6 ± 7.5	-	
Disease course, n (%)		-	
CIS	5 (7.5)		
RR	41 (61.2)		
CP	21 (31.3)		
Disease duration (years ± SD)	9.1 ± 6.3	-	
EDSS (mean ± SD)	3.9 ± 2.1	-	
	3.8^§^		
MSSS (mean ± SD)	5.4 ± 2.7	-	0.000002^#,$^
in RR group	4.6 ± 2.0		
in CP group	7.4 ± 1.3		

CIS: clinically isolated syndrome; RR: relapsing-remitting; CP: chronic progressive (including secondary progressive and primary progressive); EDSS: Expanded Disability Status Scale; MSSS: Multiple Sclerosis Severity Score; * *χ*^2^ ; ^§^ median; ^‡^ ANOVA; ^#^ ANOVA for RR vs. CP patients; ^$^ Scheffe’s post-hoc test for RR vs. CP patients; the values represent the means ± S.D.

### Ultrasound measurements

All patients and controls were examined using 7.5 MHz linear transducer for extracranial measurements and 2.5 MHz probe connected to the same ultrasound machine for transcranial analysis (Powervision 6000, Toshiba, Tokyo, Japan). Ultrasound assessments were performed in an identical fashion in patients and controls. The posture of the individual being examined by Doppler is crucial in determining the main route of cerebral outflow. Each subject was investigated in both supine and sitting positions (0° and 90°). All participants were examined by the same ultrasonographer who was blinded to the case–control selection. It is hard to be blinded to the patients with severe neurological disability, thus during the study the ultrasonographer had to examine ten more patients with high neurological disability caused by diseases other than MS (Amyotrophic lateral sclerosis, Stroke, Myasthenia gravis, Miller Fisher Syndrome), blinded to the patients diagnosis. This way we provided the blinded measurements even for the severe patients.

### Assessment of CCSVI

Both, patients and controls were examined for CCSVI according to five criteria proposed by Zamboni [2]. These criteria were: 1- Reflux (time > 0.88 seconds) in the IJVs and/or in the VVs (vertebral veins) in sitting and supine position; 2- Reflux (time > 0.5 seconds) in the deep cerebral veins; 3- Stenosis. We assessed the presence of IJV stenosis by manual measuring the cross-sectional area (CSA) of the IJV using a transverse section, above bulbus up to 15 mm above the cricoid cartilage, at the middle level (J2), adjacent to the thyroid gland. The CSA and the flow direction were measured at the end of the expiratory phase or during a short period of apnea after a normal exhalation [16]. The CSA ≤0.3 cm^2^ in the recumbent position was defined as a stenosis; 4- Flow not detected by Doppler in IJVs and/or VVs; 5- Reverted postural control of the main cerebral venous outflow pathways: a missing increase of IJV CSA in the supine position. The presence of at least two of Zamboni’s criteria in the same individual was considered positive for evidence of CCSVI.

### Assessment of small IJV

The IJV was defined as small IJV if the regional narrowing of IJV, ≤ 0.4 cm^2^, above bulbus up to 15 mm above the cricoid cartilage, at the middle level (J2), adjacent to the thyroid gland was detected. We took into account the CSA values in healthy subjects in the midcervical area [17]. If a subject demonstrated only regional narrowing of the IJV, with CSA ≤ 0.4 cm^2^, he was also considered as a small IJV positive. All individuals were examined in sitting and supine positions using the transversal access. The operator used minimal pressure over the skin to prevent compressing the vein and thereby affecting the measurement. Also, a thick layer of ultrasonic gel was used to avoid excessive pressure on the patient’s neck, which may change the shape and dimension of the IJV. The examination was repeated in randomly selected 7 patients after two weeks, and the results were 100% concordant.

### Assessment of blood volume flow (BVF)

It was previously suggested that additional assessment of hemodynamic parameters provides sufficient information to make a diagnosis of venous outflow obstruction [4]. As recommended we measured the BVF by Doppler flowmetry using the software included in the package of the ultrasound equipment (Powervision 6000, Toshiba, Tokyo, Japan). For the IJVs and VVs, the time averaged blood volume was analyzed according to protocol previously described [4]. Measurements were obtained at an identical site in supine and upright body position, the same way in patients and controls. The subjects were asked to briefly hold their breath after an exhalation, and measurements were obtained during such an episode of apnea. To avoid false positive results, we made particular efforts to avoid a compression of the vein by the transducer or neck muscles. The measurement was performed at the level of the thyroid gland for the IJVs and at the level of C5-C6 for VVs [17]. The blood flow was measured in both, IJVs and VVs separately and then calculated into total blood flow in sitting and supine position. The difference between the total BVF in supine and sitting position (∆ BVF) was calculated in both patients and controls as suggested [4,18].

### Statistical analysis

Statistical analysis was performed using Statistica software package (Version 5, StatSoft, 1997). Differences in frequency distribution between the studied groups were estimated by chi-square (*χ*2) test and confirmed by Fisher's exact test. Means of normally distributed continuous variables were compared by unpaired *t*-test or ANOVA. For variables with significantly skewed distribution, comparisons were done by nonparametric Kruskal-Wallis ANOVA. The sensitivity and specificity, AUC and CI, were determined using ROC-tool designed by Acomed statistik (Leipzig, Germany). The maximum likelihood estimation was applied in the analysis of the association between functional disability defined as categorical variable (0, EDSS < 6 or 1, EDSS ≥ 6) and small IJV, expressed in terms of OR and 95% confidence interval (CI). In multivariate regression analysis, which included the factors that were significantly different in-between groups and kept covariates in the model if the p of the model was <0.001. Differences with two-tailed alpha-probability p ≤ 0.05 were considered significant. To correct for multiple comparisons, a conservative Type I error level of 0.01 was used to assess significance of chi-square test and logistic regression results. Statistical power of the study for the effect of sIJV on functional disability was calculated using the PS (v3.0.43) [19].

## Results

The characteristics of the patients and controls are presented in Table 1. Demographic characteristics of the MS patients and the controls were not significantly different (Table 1). Chronic progressive (CP) patients (including secondary progressive, SP and primary progressive, PP) had significantly higher Multiple Sclerosis Severity Scores (MSSS) compared to relapse-remitting (RR) patients (p = 0.000002).

### CCSVI in MS patients and controls

**Figure 1:**
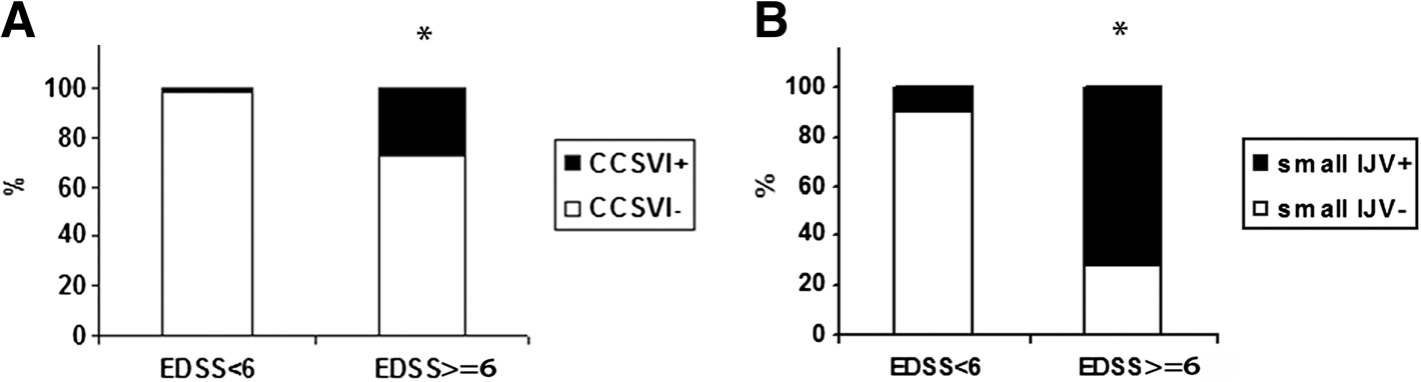
**Prevalence of CCSVI and small IJV as a function of EDSS (EDSS < 6 vs. EDSS ≥ 6).** Shows prevalence of **A)** CCSVI, CCSVI + n = 7/25 with EDSS ≥ 6, (*χ^2^, p = 0.002), and **B)** small IJV, sIJV + n = 15/ 25 MS patients with EDSS ≥ 6 (*χ^2^, p = 0.00001) in MS patients categorized by functional disability (EDSS < 6 vs. EDSS ≥ 6). EDSS: Expanded Disability Status Scale; CCSVI: chronic cerebrospinal venous insufficiency; small IJV: small internal jugular vein.

Out of 67 MS patients, 8 (11.9%) were positive for CCSVI according to the criteria described in the Experimental Methods. None of the controls were CCSVI positive. The prevalence of CCSVI according to different clinical courses of MS (CIS, RR and CP) is shown in Table 2. CCSVI was significantly more prevalent among the chronic progressive patients (CP) compared to the non-progressive patients (CIS and RR) (23.8% vs. 6.5%, two-tailed *χ*^2^ p = 0.043). CCSVI was also significantly more common among the patients with severe MS (EDSS ≥ 6) (n = 7 were CCSVI positive out of 25 patients with EDSS ≥ 6, p = 0.002, Figure 1) and was associated with a significantly higher MSSS (p = 0.008) (Table 2). Multivariate logistic regression analysis adjusted for gender and disease duration revealed that CCSVI was not a significant independent predictor of MS disease severity (EDSS ≥ 6) (p = 0.06). However, disease duration was an independent predictor of MS disease severity (OR = 1.3, 95% CI: 1.1-1.6; p = 0.0003).

**Table 2:** Characteristics of patients with MS according to CCSVI status

**Parameter**	**Patients (n =67)**		**Controls (n =21)**		**p**
	**CCSVI-**	**CCSVI+**	**CCSVI-**	**CCSVI+**	
n (%)	59 (88.1)	8 (11.9)	21(100)	0 (0)	0.1*
Female/male (%)	49.2/50.8	50.0/50.0	33.3/66.7	0/0	ns*
Age at onset (years ± SD)	27.5 ± 7.2	29.1 ± 9.7	-		0.6^#^
Disease duration (years ± SD)	8.4 ± 5.9	14.4 ± 6.5	-		0.01^#^
Disease course, n (%)			-		0.12*
CIS	5 (8.5)	0			
RR	38 (64.4)	3 (37.5)			
CP	16 (27.1)	5 (62.5)			
Total	59 (100)	8 (100)			
MSSS (mean ± SD)	5.2 ± 2.2	7.4 ± 1.4	-		0.008^#^
EDSS (mean ± SD)	3.6 ± 2.0	5.85 ± 1.8			0.007^#^

CCSVI: chronic cerebrospinal venous insufficiency; CIS: clinically isolated syndrome; RR: relapsing-remitting; CP: chronic progressive (including secondary progressive and primary progressive); MSSS: Multiple Sclerosis Severity Score; EDSS: Expanded Disability Status Scale; * *χ*^2^; ^#^ ANOVA; values represent the means ± SD; ns: non-significant.

### Small IJV in MS patients and controls

We found that 28.4% (n = 19) of the patients and 28.6% (n = 6) of the controls had at least one small internal jugular vein (CSA ≤ 0.4 cm^2^) (Table 3). The majority of the small IJVs were detected on the left side in both the patients and the controls (66.1% and 68.1%, respectively). Among the patients with small IJVs the 36.84% were also CCSVI-positive. The prevalence of small IJVs differed significantly according to different clinical courses of MS (p = 0.0002), with the highest prevalence in the CP group (68.4%, all SP). Small IJVs were significantly more common among the patients with severe MS (EDSS ≥ 6) (n = 15 were sIJV positive out of 25 patients with EDSS ≥ 6, p = 0.00001, Figure 1). The mean MSSS was significantly higher among the patients with small IJVs (p = 0.002, Table 3). Multivariate logistic regression analysis adjusted for gender, disease duration and the side where the small IJV was located, has revealed that the patients with small IJVs were 8.9-fold more likely to develop a severe form of MS (EDSS ≥ 6) (adjusted OR = 8.9, 95% CI: 1.8-45.6, p = 0.007). The power of the study to detect an association between small IJVs and EDSS ≥ 6 with OR = 8.9 was 89% (calculated retrospectively with an increased stringency of the Type I error level at 0.01). We also performed a multivariate regression with MSSS as a clinical parameter of disease severity (the cut-off value was taken as the median). Patients with MSSS ≥ 5.4 (median) were considered to have severe MS. Again, the results revealed a significant adjusted association between the presence of small IJVs and MS severity (OR = 4.7, 95% CI: 1.4-16.5, p = 0.012).

**Table 3:** Characteristics of patients with MS according to small IJV status

**Parameter**	**Patients (n = 67)**		**Controls (n = 21)**		**p**
	**small IJV-**	**small IJV+**	**small IJV-**	**small IJV+**	
n (%)	48 (71.6)	19 (28.4)	15 (71.2)	6 (28.8)	ns*
Female/male (%)	45.8/54.2	52.6/47.4	40/60	16.7/83.3	ns*, ns*
Age at onset (years ± SD)	27.4 ± 7.1	28.0 ± 8.7	-		0.8^#^
Disease duration (years ± SD)	7.2 ± 5.4	13.8 ± 6.0			0.00004^#^
Disease course, n (%)			-		0.0002*
CIS	5 (10.4)	0 (0)			
RR	35(72.9)	6 (31.6)			
CP	8 (16.7)	13 (68.4)			
Total	48 (100)	19 (100)			
MSSS (mean ± SD)	4.8 ± 2.1	6.8 ± 2.2	-		0.002^#^
EDSS (mean ± SD)	3.2 ± 1.8	5.6 ± 1.9			0.00003^#^

Small IJV: small internal jugular vein (CSA ≤ 0.4 cm^2^); CIS: clinically isolated syndrome; RR: relapsing-remitting; CP: chronic progressive (including secondary progressive and primary progressive); MSSS: Multiple Sclerosis Severity Score; EDSS: Expanded Disability Status Scale; * *χ*^2^; ^#^ ANOVA; the values represent the means ± SD; ns: non-significant.

### Blood volume flow

**Figure 2:**
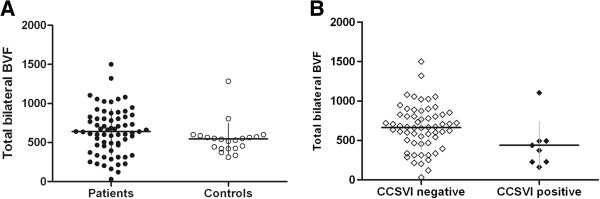
**Total bilateral blood volume flow (BVF).** Scatterplot of the total BVF (mL/min) relative to the mean BVF among **A)** patients with MS and controls, p = 0.26; **B)** CCSVI-positive and CCSVI-negative MS patients, p = 0.07.

**Figure 3:**
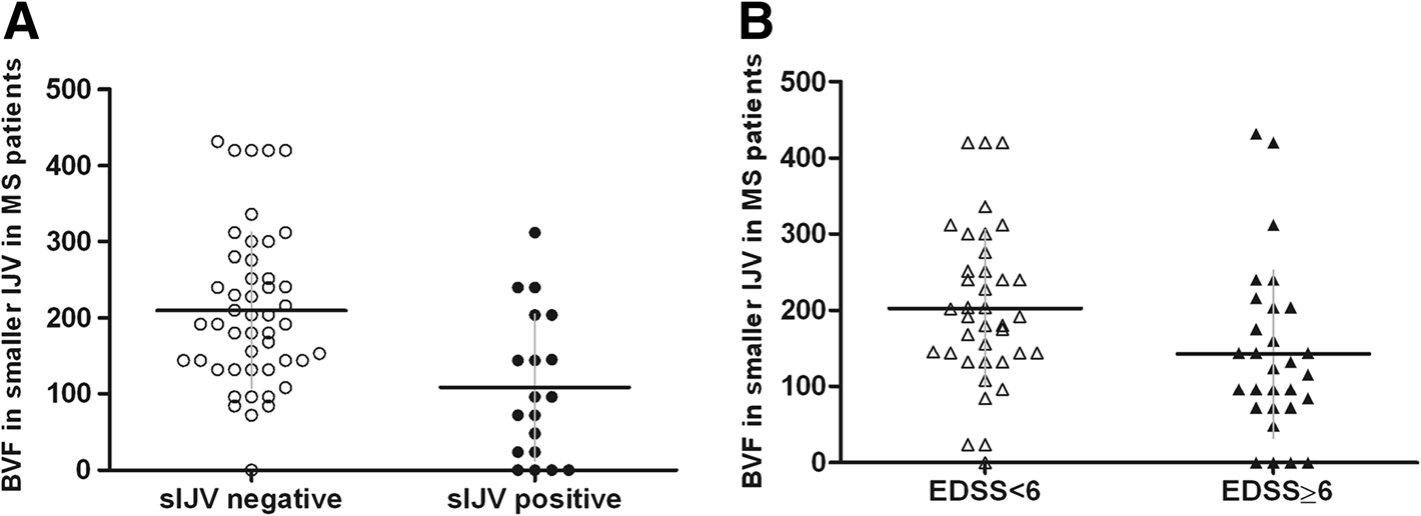
**Total bilateral blood volume flow (BVF) in the smaller IJV of MS patients.** Scatterplot of the BVF (mL/min) relative to the mean BVF in the smaller IJV in the supine position among **A)** small IJV-positive MS patients and small IJV-negative MS patients, p = 0.001*; **B)** MS patients with EDSS < 6 and EDSS ≥ 6, p = 0.04*.

The mean total bilateral blood volume flow (BVF) (mL/min) in the IJVs and VVs with the subject in either a supine or sitting position was not significantly different between the patients vs. the controls (supine: 639.17 ± 306.05 vs. 548.47 ± 223.16, respectively, p = 0.26; sitting: 492.55 ± 232.79 vs. 411.95 ± 208.63, respectively, p = 0.22) (Figure 2A). The mean value of difference in the BVF between the supine and upright position (∆ BVF) was not significantly different between patients with MS and controls (p = 0.8). Furthermore, the BVF was not significantly different between the small IJV-positive and -negative patients (supine: 531.39 ± 346.15 vs. 656.20 ± 259.55, respectively, p = 0.08, sitting: 423.27 ± 223.46 vs. 497.15 ± 229.61, respectively, p = 0.21). The total BVF was not significantly lower among the CCSVI-positive compared to the CCSVI-negative MS patients (supine: 439.71 ± 320.96 vs. 664.21 ± 300.18, respectively, p = 0.07; sitting: 404.00 ± 161.11 vs. 481.24 ± 233.74, respectively, p = 0.46) (Figure 2B). The mean BVF measured in the smaller IJV in all the patients was significantly decreased in the small IJV-positive compared to small IJV-negative patients (supine: 108.69 ± 96.06 vs. 209.56 ± 109.76, respectively, p = 0.001; sitting: 70.40 ± 68.67 vs. 129.00 ± 109.66, respectively, p = 0.03) (Figure 3A). The patients with severe MS (EDSS ≥ 6) had decreased BVF in the smaller IJV compared to the patients with EDSS < 6, a finding that was significant only in the supine position (142.56 ± 118.81 vs. 202.66 ± 106.91, respectively, p = 0.04) (Figure 3B). The sensitivity and specificity of BVF, in the smaller IJV in the supine position, as a predictor of either small IJV or EDSS ≥ 6, exhibited values below 50% (AUC: 0.25, SE: 0.06 and 95% CI: 0.15-0.38; and AUC: 0.32, SE: 0.07 and 95% CI: 0.2-0.45, respectively).

## Discussion

In this study, we investigated the possibility of a causal association between CCSVI and MS, and small IJVs and MS, relationships that had not been previously examined in a South-Eastern European population. Neither CCSVI nor sIJVs prevalence was significantly different between patients with MS and controls. The main findings of the study were that both CCSVI and small IJVs seem to influence or follow MS severity. For the first time, in this study, the small IJVs were revealed as an independent factor associated with MS severity.

A subset (11.9%) of MS patients fulfilled at least two criteria for CCSVI. The CCSVI-positive patients presented with significantly longer durations of MS, and their EDSS and MSSS scores were significantly higher than those of the CCSVI-negative patients. Prevalence of CCSVI was higher among the chronic progressive patients (SP and PP) compared to non-progressive ones. This finding is in line with two recent studies in which patients with the progressive form of MS had a significantly higher CCSVI prevalence [3,20]. In accordance with these studies [3,20], we did not find a significant independent association between CCSVI and MS disease severity. Several studies on patients with clinically definite MS have reported that over 50% of these patients were CCSVI-positive [2,3,7,21], whereas others have found an absolute lack of a positive correlation [4,22]. However, the findings in the controls have been more homogenous. We did not find two or more CCSVI criteria among our control subjects, similar to others [4,9]. The main hypothesis supported by our data and by the results of the largest published studies [3,23] is that CCSVI does not have a primary causative role in MS but instead plays a contributing role or is a consequence of the disease and long MS duration.

There are weaknesses in this and other studies, which are mainly attributable to the methodological approaches employed. Extracranial color-Doppler high-resolution examination, which we used in this study, has been proposed as the most appropriate method to observe dynamic changes in venous outflow [24]. However, a large, recent study using catheter venography demonstrated a prevalence of pathologies in specific veins of up to 81.7% [23], much higher than the levels detected by Doppler. It has been suggested that Doppler can detect the competence of the IJV valve, the cross-sectional area in relation to a change in posture, duplex-derived flowmetry and anomalous morphology [24], but it is likely that there are other methods that allow a more precise detection of morphological abnormalities. Thus, we must be cautious in drawing conclusions because there is a possibility that we were unable to detect all venous pathologies, both in the patients and the controls. Although it has recently been suggested that Doppler has limited diagnostic value for diagnosing venous morphological changes [25], others claim that the inter-rater reproducibility supports its use for diagnostic purposes in multicenter studies [8,26]. Ultrasound technology, which is widely used in this field, is promoted by the revised protocol for CCSVI screening [27] and is particularly advantageous due to its noninvasive nature. The methodological heterogeneity of detection of venous morphological changes might, at least in part, explain different conclusions of the previous studies.

Another possible limitation is the use of standard CCSVI criteria. Five ultrasound criteria were proposed by the founders of the CCSVI hypothesis [2], but these have yet to be validated against a criterion standard [28]. For the third criterion, we defined IJV stenosis as CSA ≤ 0.3 cm^2^[2]. Initially, the cutoff for stenosis was defined as a local CSA reduction of ≥50% [9], a threshold that was adopted by other researchers [4]. In the same year, the same group proposed a stenosis reference value of a CSA ≤ 0.3 cm^2^[2], which was again accepted by other researchers [22]. This reference value (CSA ≤ 0.3) arose somehow from the original proposal of a CSA ≤ 0.4 cm^2^ as the cutoff for small veins that are inadequate for catheterization [11]. Discrepancies in definition of stenosis have been previously discussed [22]. Nevertheless, some discrepancies between the studies could also be linked to the anatomical level of CSA measurement [2-4,6]. In the present study, the sonographer insonated the IJV at all levels (J1, J2 and J3) and measured the CSA at the middle level (J2), adjacent to the thyroid gland, as proposed in the revised protocol [27]. These issues should be resolved by the updated protocol [27], which is much more precise than the previous definition of the CCSVI criteria.

In addition to CCSVI, we investigated the relationship between small IJVs (CSA ≤ 0.4 cm^2^) and MS. This is the first study to examine this potential association. Similarly to CCSVI, the prevalence of small IJVs was not different among the MS patients compared to the controls; however, small IJVs were significantly more common among the patients with progressive form of the disease. The most important finding was that small IJV represents an independent factor associated with MS severity. It has been suggested that venous malformations may have a genetic etiology, and both environmental and genetic factors could play a role in venous pathophysiology [29,30]. Asymmetries and inborn anatomical differences have been observed in the truncular venous system [31,32], particularly during catheterization/interventional procedures in different groups of patients [11,33-36]. A narrowing of IJVs with a CSA < 0.4 cm^2^, which are defined as small veins, was observed in 23% of IJVs in different cases admitted to the intensive care unit [11]. In our study, 28% of both MS patients and controls were found to be small IJV-positive, which is in line with previous studies. Majority of the small IJVs were detected on the left side, both in MS patients and the controls, similar to findings in a general population of adult outpatients [37] and in the majority of CCSVI lesions [23]. However, a possibility exists that some vein narrowing could be caused by muscular compression [38] or an asymmetric pattern of intracranial sinuses [39].

The EDSS and MSSS scores were significantly higher among small IJV-positive patients compared to those presenting with normal veins. Our results are in agreement with a recent study that has revealed longer disease durations and higher EDSS scores in the group of MS patients with >80% jugular narrowing compared to the group with less narrowing [40]. We found a significantly higher prevalence of the patients with EDSS ≥ 6 in the small IJV-positive group. EDSS ≥6 is a stringent cut-off value, but we chose this criterion because, according to the Kurtzke scale [15], an EDSS of 6–10 indicates severe disease (the patient requires support to walk). Nonetheless, we also performed a multivariate regression and used the MSSS as a clinical parameter of disease severity (with the cutoff at the median). The results confirmed a significant independent association between the presence of small IJVs and disease severity. It is difficult to compare our results to those of the others, since no other trials have investigated small IJVs or even IJV segmental stenosis as a single parameter potentially associated with MS. A higher prevalence of small IJVs in chronic progressive patients (68.4%) is in line with previous finding that IJV stenosis is more frequent among SP and PP compared to RR patients [2]. An association between late-stage MS and venous stenosis has also been observed [41]. Similarly, in our study MS patients with small IJVs had longer disease duration. Taken together, these results suggest that both subtypes of truncular IJV malformation (IJV stenosis and small IJVs) may influence MS severity.

The BVF data certainly provide a comprehensive analysis of venous outflow obstruction. As suggested by others [4], we included the BVF measurement to improve evaluation of hemodynamic effects of any suspected cerebrocervical venous congestion. The total BVF of the subjects in the supine position in our study was similar to that reported previously [42]. However, according to one suggested interpretation of the AUC (area under the curve) [43], the BVF in the IJV in this study was a poor predictor of either the presence of small IJVs or MS severity. Still, the methodological differences can be a question in the BVF assessment. One of the shortcomings in this study was the measurement of the BVF only in J2 level, which might be the explanation of the absence of difference between patients and controls. According to recent study it should be measured at J1 where it is the highest, in both patients and controls [16]. The others performed the measurement of IJV BVF as apical as possible in the upper region of the neck [4]. Besides, BVF is a parameter that exhibits interindividual variations; therefore, the relatively small number of measurements in our study may account for the lack of significance of this result.

The IJVs are considered to be the main pathways of cerebral blood drainage. The IJVs shows physiological variations of its diameter. The wide anatomical variability and varying degrees of jugular and non-jugular venous drainage have been shown in the healthy volunteers [42]. The pathophysiological significance of changes in IJVs, either in its anatomy or function in blood drainage has not been completely understood. It has recently been suggested that both cardiovascular autonomic nervous system dysfunctions and dysfunctions of the noradrenergic neurons in the cerebral venous system could result in an impairment of cerebral autoregulation and in reduction of vascular tone, which would in turn promote venous closure [44]. Also, IJVs drain the blood, which was shown to contain metabolically active substances. It was suggested that early activation of the l-arginine/NO pathway, which accompanies the release of vasoactive peptides happens in the migraine [45]. It was detected by analyzing the internal jugular venous blood of patients. Further studies should investigate metabolic changes in the brain by analysis of blood drained through jugular pathways, as it could shed new light on the both, MS associated changes and possible pathophysiological role of small IJVs in MS. According to our results and recent finding on magnetic resonance venography [40], small IJVs could have pathophysiological significance in MS. Still, the measurable hemodynamic effect were detected only in vessels with narrowing >80%, the group with the highest frequency of secondary progressive MS patients [40]. The small IJVs could be partially the primary phenomenon, as they are present in control group, but also could be in part the secondary one that develops as a consequence of MS. Further studies are needed to replicate these results and to resolve whether small IJVs are a cause or a consequence of MS.

## Conclusions

We conclude that the presence of small IJVs (CSA ≤ 0.4 cm^2^) may be an independent cofactor in the multifactorial mechanism of MS pathogenesis. The presence of small IJVs is associated with the increased probability of a severe clinical course of the disease. This finding may be important for the future treatment of MS, but it first must be validated and replicated by other studies, especially with severely disabled patients.

## Competing interests

Authors declare no competing interests.

## Authors’ contributions

ZK contributed to study design, contributed to Doppler data acquisition, interpretation of findings and manuscript preparation. MZ performed the statistical analysis, data interpretation and manuscript preparation. TL contributed to patient recruitment and clinical data acquisition. AS contributed to interpretation of findings and manuscript preparation. RR contributed to clinical data acquisition and study coordination. ED contributed to study design and its coordination, and helped to draft the manuscript. All authors read and approved the final manuscript.

## Pre-publication history

The pre-publication history for this paper can be accessed here:

http://www.biomedcentral.com/1471-2377/13/90/prepub
